# *Scutellaria baicalensis* and its flavonoids in the treatment of digestive system tumors

**DOI:** 10.3389/fphar.2024.1483785

**Published:** 2024-11-25

**Authors:** Kangning Zhao, Jinlong Zhang, Lin Zhou, Zhen Sun

**Affiliations:** ^1^ The First Clinical Medical College, Shandong University of Traditional Chinese Medicine, Jinan, China; ^2^ The Second Gastroenterology Department, Affiliated Hospital of Shandong University of Traditional Chinese Medicine, Jinan, China

**Keywords:** *Scutellaria baicalensis*, baicalein, baicalin, tumor, TCM, nature products

## Abstract

*Scutellaria baicalensis* has been used for the treatment of digestive system disorders for thousands of years in China and other regions. Modern research have revealed its therapeutic efforts in digestive system tumors. Thus, to review the updated progress of *S. baicalensis* and its main flavonoids in the treatment of digestive system tumors in the past 10 years, this article summarized the therapeutic effect and molecular mechanisms of *S. baicalensis* and its 5 flavonoids on tumors in oral cavity, esophagus, stomach, colon, liver, pancreas by inhibiting tumor cell proliferation, inducing autophagy, stimulating immune response, and increasing drug sensitivity. In conclusion, *S. baicalensis* and its flavonoids could be applied to treat digestive system tumors with different type of methods.

## 1 Introduction

Digestive system tumors, such as oral squamous cell carcinoma (OSCC), laryngeal cancer, esophageal cancer (EC), gastric cancer (GC), colorectal cancer (CRC) occurring in the tract and hepatocellular carcinoma (HCC), pancreatic cancer (PC) occurring in the glands, are a series of tumors with high morbidity and mortality worldwide. Statistically, CRC (1,880,725/915,880), GC (1,089,103/768,793), and HCC (905,677/830,180) rank the 3rd, 5th, and 6th of the number of new cases and the 2nd, 4th, and 3rd of the number of deaths of cancers per year globally, respectively (Sung H et al., 2021). The related cases accounted for 26% of global cancer incidence yet at least 35% of mortality in 2018, which suggests poor survival outcomes (Arnold M et al., 2020). After years of development, many treatment modalities such as chemotherapy, radiotherapy and surgery are now available. However, these tumors are very unremarkable in the early stages due to the depth of the organs, insensitivity of the visceral nervous system, etc., which makes their timely diagnosis difficult, leading to late treatment and plagues clinical care. In recent years the adjuvant role of traditional Chinese medicines (TCMs) such as *Scutellaria baicalensis* in the treatment of a variety of tumors has been increasingly validated, bringing more opportunities for the improvement of clinical efficacy and the development of novel drugs (Ganguly R et al., 2022).

*Scutellaria baicalensis* (Huang qin), the dried root of *S. baicalensis* Georgi, family Labiatae, is one of the most commonly used herbs for the treatment of digestive disorders in East Asia, South Asia, and Turkey accompanied with a long history of application and increasing use (Cheng CS et al., 2018). In ancient China, many medical texts classified it in the treatment of lung and stomach diseases, stating that it can “clear heat and dry dampness, diarrhea and detoxification, stop bleeding, and protect the foetus” and is often used to ameliorate fever, diarrhea, vomiting, and jaundice, which are also the symptoms of many malignant tumors, hinting at its excellent role in respiratory and digestive disorders (Lin HH et al., 2024). In particular, the use of *S. baicalensis* in the treatment of COVID-19 proved its unique effect (Liu J et al., 2022; Dinda B et al., 2023; Zhang et al., 2021a). With reference to the ancient literature on its indications, the role of *S. baicalensis* and its bioactive ingredients in digestive tumors is gradually being clarified through modern pharmacological studies. However, within our knowledge, its interventional role in digestive tumors has not been specifically summarized in recent years. Therefore, this review is based on the search in electronic databases such as PubMed, Web of Science, Google Scholar and China National Knowledge Infrastructure (CNKI),whose keywords are “*S. baicalensis*”, “baicalein”, “baicalin”, “wogonin”, “wogonin”, “wogonin” and “wogonin”. “wogonin”, “wogonoside”, “oroxylin-A″, “cancer” and others. The experimental studies of *S. baicalensis* and its flavonoids in the treatment of digestive system tumors were compiled and summarized from 2014 to 2024, in an attempt to show the research trends in this field in recent years, and thus provide reference for the experimental research and clinical application of TCM in the prevention and treatment of digestive system tumors. As can be seen, most of the relevant studies are experiments with different doses of flavonoids and cultured tumor cells, using pooled colony assay to study the proliferative ability of tumor cells and Transwell assay to measure the invasive ability of the cells. Few *in vivo* experiments were performed on animals transplanted with tumor cells by oral administration or injection of the ingredients to measure changes in tumor volume and molecular expression. Techniques such as Western blot and ELISA are used to detect the expression of relevant proteins and molecules.

## 2 Application of *Scutellaria baicalensis* and its flavonoids in digestive system tumors

First recorded in the Shennong Ben Cao Jing (Classic of the Materia Medica of the Divine Husbandman) in China in the 2nd century B.C., *S. baicalensis* is said to have the efficacy of “treating all kinds of fever, jaundice, diarrhea, edema, and sores”. Numerous subsequent medical texts distinguish between “solid one (Ku qin, growth years >3)” and “hollow one (Zi qin, growth years ≤3)”. The latter has been documented to be more effective in digestive complaints and dominates the current market distribution (Zhan X et al., 2021).

The vigorous development of modern pharmacology in recent years, especially the gradual clarification of disease pathology and bioactive ingredients of herbs, has also provided new references beyond clinical experience for the application of TCM including *S. baicalensis*. So far, there have been at least 132 flavonoids, 17 hydrocarbons, 17 terpenoids, 18 amino acids, 30 organic acids, 6 esters, 7 aldehydes and ketones, 8 phenylpropanoids, 9 alkaloids, 10 sugars, 11 alcohol components, and 3 steroidal components were identified from more than 2000 compounds of *S. baicalensis* (Huang LY et al., 2023). Among them, flavonoids such as baicalein, baicalin, wogonin, wogonoside and oroxylin-A attracted the most attention because of their high concentration and excellent effects. Studies have confirmed that *S. baicalensis* and its flavonoids have different degrees of interfering effects on various pathways of the digestive system, which are closely related to tumor development (Dmitrieva A et al., 2023; Tuli HS et al., 2023; Jang JY et al., 2023; Chmiel and Stompor-Gorący, 2023).

Baicalein (5,6,7-trihydroxyflavone, C_15_H_10_O_5_), the flavonoid that has been studied most, is widely known for its function against COVID-19 (Su HX et al., 2020). In addition, it has been used for cardio protection and to help overcome chemotherapeutic drug resistance in tumors successfully (Yang Q. et al., 2024; Wang T. et al., 2024; Chen T. et al., 2024).

Baicalin (baicalein-7-O-glucuronide, C_21_H_18_O_11_) is metabolized to baicalein in animals (Kang MJ et al., 2014). The Chinese Pharmacopoeia sets baicalin content of not less than 8% as the standard for qualification of herbs. It has been shown to have good antioxidant, anti-inflammatory and antitumor effects, especially in the nervous system (Liu K. et al, 2024; Liu ZSJ. et al, 2024; Wang H. et al, 2024; Noor S et al., 2024).

Wogonin (5,7-dihydroxy-8-methoxyflavone, C_16_H_12_O_5_), has demonstrated its value in improving hepatic metabolism and treating colitis (Yamada Y et al., 2022; Ye Q et al., 2024).

Wogonoside (5,7-dihydroxy-8-methoxyflavone, C_22_H_20_O_11_) has previously received widespread attention for its therapeutic effects on respiratory and cardiac inflammation (Feng W et al., 2023; Yu X et al., 2024).

Oroxylin-A (5,7-dihydroxy-6-methoxyflavone, C_16_H_12_O_5_) is a potent antioxidant capable of exerting anti-inflammatory and hepatoprotective effects (Liu T. et al., 2024; Cho W et al., 2023; Zhu J et al., 2023).

**FIGURE 1:**
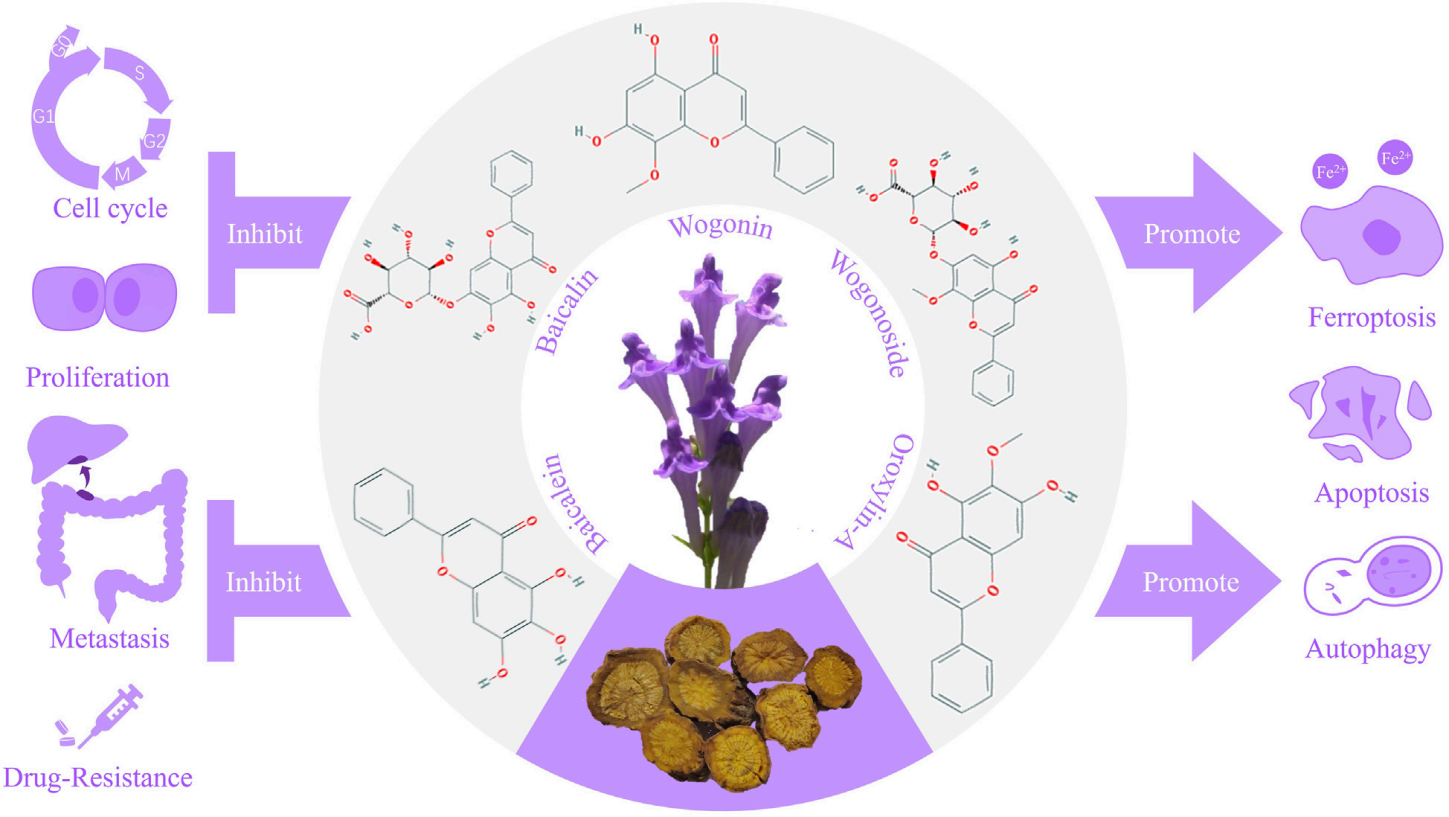
Mechanisms of *Scutellaria baicalensis* and the main flavonoids in the treatment of digestive system tumors.

Besides, other flavonoids of *S. baicalensis*, such as Scutellaria flavone Ⅰ and Scutellarin, functions in the treatment of digestive system tumors as well. The (Figure 1) showed the characteristics of *S. baicalensis* and structures of the main flavonoids, along with the mechanisms involved in the treatment of digestive system tumors.

## 3 Current production of *Scutellaria baicalensis*

Botanical sources of *S. baicalensis* and substitutes include primarily northern *S. baicalensis* (such as *Scutellaria viscidula* Bge., *Scutellaria rehderiana* Diels) and southwestern *S. baicalensis* (such as *Scutellaria amoena* C.H. Wright, *Scutellaria hypericifolia* Levl., *Scutellaria likiangensis* Diels and *Scutellaria tenax* W.W. Smith var. patentipilosa (Hand.-Mazz.) C.Y. Wu). Metabolomics studies have shown that the accumulation of primary metabolites, such as flavonoids, of *S. baicalensis* showed significant differentiation depending on the latitude and longitude of the growing site (Sun et al., 2023b). Using MaxEnt and ArcGIS systems to predict the ecological suitability of *S. baicalensis*, it was found that the main suitability zones in China were distributed in subalpine evergreen coniferous scrub, norm temperate and temperate montane coniferous forests, as well as temperate steppe-zed shrub deserts (Xu et al., 2024). However, the analysis of *S. baicalensis* and wined *S. baicalensis* using HPLC fingerprinting technique showed that the contents of the main components of several dried herbs of the same herb originating from different places were basically the same, and the large-scale application of *S. baicalensis* was still secured (Xiong Y et al., 2018). In addition, it was experimentally confirmed that SSR molecular marker technology based on the combination of 3 groups of primers could reliably identify the genetic material of *S. baicalensis* from different origins (Liu et al., 2021). Due to its wide geographical distribution and different concoctions, the pharmacological effect of *S. baicalensis* dried herbs has obvious differences. For example, one study reported that the constituents of the dried herbs had high similarity but significant geographic variations among homologous Chinese herbs (Liu et al., 2023c). Another recent study found that *S. baicalensis* from Gansu, Shandong and Henan provinces had the highest flavonoid content and best quality after HPLC determination in various genuine producing areas in China (Zheng Y. et al., 2023). Interestingly, besides baicalein, oroxylin-A and baicalin, differences in antioxidant activity *in vitro* can also be a useful way to differentiate between different sources of *S. baicalensis* (Yuran et al., 2024). From these, it can be seen that there are already clear criteria for its botanical sources, which creates a convenient way for subsequent research and use.

## 4 Therapeutic role of *Scutellaria baicalensis* in digestive system tumors

### 4.1 Oral squamous cell carcinoma and laryngeal cancer

OSCC is the most common and fatal malignant tumor in the head and neck region, which is prone to metastasis through the lymphatic system to become a systemic cancer (Tandon A et al., 2023). And the incidence of laryngeal cancer is increasing worldwide (Nocini R et al., 2020). The therapeutic effects of flavonoids from *S. baicalensis* on them have been recognized recently (Table 1).

**TABLE 1:** Flavonoids of *Scutellaria baicalensis* in the treatment of oral squamous cell carcinoma and laryngeal cancer.

Name	Dose	Subjects	Mechanism	Effect	Reference
Baicalein	30, 60, 120 μM 24, 48, 72h; 30 mg/kg/2 days ip for 21 days	SCC25 cells; SCC25 cells xenograft BALB/c mice	cleaved caspase-9↑ cleaved caspase-3↑ cleaved PARP-1↑ Sp1↓ p50↓ p65↓	Promote apoptosis Induce cell cycle arrest at G0/G1 phase	Gao Z et al. (2020)
	12.5, 25, 50, 100, 200 μmol/L for 48 h	CAL27 cells	ROS↑MMP↓	Promote apoptosis Induce cell cycle arrest at S phase	Shi NX et al. (2023)
	25, 50, 100 μM for 4 h	CAL27 cells	ROS↑ Bax↑ cleaved PARP↑ Bcl-2↓	Promote autophagy ang apoptosis	(Liu B et al., 2017)
	10, 30, 100, 300 μmol/L for 48 h	AMH-HN-8 cells	Cyto-c↑ Bax↑ cleaved caspase-3↑Bcl-2↓	Inhibit proliferation and invasion Promote apoptosis	Wang et al. (2023a)
	200 μmol/L for 24 h	Hep-2 cells	Beclin-1↑ LC3Ⅱ↑ miR-449a↑ p62↓ LC3Ⅰ↓ HDAC1↓	Inhibit proliferation Promote apoptosis	Wang et al. (2023b)
Baicalin	100, 150, 200 mg/L for 1 w	CAL27 cells	BAX↑ IL-18↓ IL-1β↓ p-JAK2/JAK2↓ p-STAT3/STAT3↓ PCNA↓ MMP-9↓	Inhibit proliferation and invasion Promote apoptosis	Dai Q et al. (2024)
	10, 20, 30, 40, 50, 100 μ for 24 h	CAL27 cells	E-cadherin↑ vimentin↓ Snail↓ Notch↓ JAG1↓	Inhibit EMT, cell viability and proliferation Promote apoptosis	Wang et al. (2023a)
	5, 10, 20, 40, 60 μM for 24 h	CAL27 cells	Fe^2+^↑ MDA↑ ROS↑ GSH↓	Inhibit EMT Promote ferroptosis	Wen Z et al. (2024)
Wogonin	25, 50, 100, 200, 400 mg/L for 24, 48, 72 h	HN-6 cells	Bax↑ Bel-2↓	Inhibit proliferation Promote apoptosis Induce cell cycle arrest at G0/G1 phase	Dong WX et al. (2017)

#### 4.1.1 Baicalein

Recent studies illustrate that baicalein directly inhibits OSCC growth in several methods. Specificity protein 1 (Sp1), a zinc finger type-transcription factor, is involved in multiple behaviors of tumor cells such as growth, survival and apoptosis (Pan J et al., 2024). Compared with that cells treated with DMSO, Western blot analysis showed that G0/G1 phase cycle arrest and apoptosis induced by baicalein in OSCC cell lines SCC25, CAL27, and HSC3 cells was accompanied by elevated levels of cleaved caspase-9, cleaved caspase-3, cleaved PARP-1 and decreased levels of Sp1, p50, and p65. In addition, silencing Sp1 was able to inhibit NF-κB activity. Anatomical and immunohistochemical analyses of baicalein-treated xenograft mice showed the same changes of SCC25 cells. This study provides a more comprehensive reference for the inhibitory proliferative and pro-apoptotic effects of baicalein on OSCC cells through Sp1 (Gao Z et al., 2020). Besides, baicalein induced S-phase arrest and apoptosis in tongue squamous cell carcinoma CAL27 cells through dose-dependent upregulation of reactive oxygen species (ROS) and downregulation of MMP, which could be reversed by ROS inhibitors, suggesting a role for activation of mitochondrial oxidative stress pathway (Shi NX et al., 2023). Consistent with this, baicalein induced autophagy and apoptosis in CAL27 cells through dose-dependent upregulation of ROS, Bax, cleaved PARP and downregulation of Bcl-2, which could be reversed by the ROS inhibitor NAC as well. Interestingly, pharmacological or genetic blockade of autophagy enhanced baicalein-induced apoptosis. It is reasonable to assume that inhibiting ROS-dependent autophagy and thereby enhancing baicalein effects is a viable therapeutic strategy for OSCC (Li B et al., 2017).

Baicalein is also known to be therapeutic for laryngeal cancer. MicroRNAs (miRNAs) are a class of endogenous non-protein-coding RNAs, among which miRNA-125b-5p and miRNA-499 widely regulate the growth and apoptosis of a variety of tumors such as neck squamous cell carcinoma, chordoma, and HCC. Many studies have shown that they can act as valuable tumor suppressors (Yuan L. et al., 2023; Huo X et al., 2023; Jiang JK et al., 2023). In connection with this, HDAC1 has been confirmed to be a downstream target of miRNA- 499a against malignant tumors and upregulated in laryngeal cancer cells (Ishikawa D et al., 2019; Chistiakov DA et al., 2017). Wang et al. (2023c) found that baicalein dose-dependently inhibited the proliferation and invasion of laryngeal carcinoma AMC-N-8 cells, inducing apoptosis by inhibiting interferon regulatory factor 4 (IRF4) thereby activating pro-apoptosis-related proteins Cyto-c, Bax, cleaved caspase-3 and inhibiting apoptosis inhibitory protein Bcl-2 in laryngeal cancer cells. Then, miR-125b-5p inhibitor reversed the inhibitory effect of baicalein, which confirmed the target (Wang et al., 2023a). Another study of them showed that autophagy induced by baicalein is accompanied with upregulated miR-449a and downregulated HDAC1 expression in Hep2 cells. The autophagy inhibitor 3-MA partially deregulated the inhibitory effect, confirming that baicalein inhibits laryngeal cancer development via autophagy in the miR-499a/HDAC1 axis (Wang J et al., 2023b). Together, these experiments demonstrate the role of miRNA-mediated autophagy and apoptosis in baicalein’s anti-laryngeal cancer process.

#### 4.1.2 Baicalin

Dysregulation of the JAK2/STAT3 pathway, an important intracellular cascade, promotes tumor development (Kohal et al., 2024). A recent study measured IL levels by using ELISA and JAK2/STAT3 pathway-associated proteins’ level by using Western blot. Baicalin induced apoptosis and inhibited cell proliferation, invasion in CAL27 cells, accompanied by BAX upregulation and IL-18, IL-1β, p-JAK2/JAK2, p-STAT3/STAT3, PCNA, and MMP-9 downregulation. It can be seen that baicalin also counteracts OSCC by inhibiting the JAK2/STAT3 pathway, which works both in OSCC and CRC. Unfortunately, these inferences were not verified by further animal experiments (Dai Q et al., 2024). The Notch signaling pathway is an intercellular communication pathway that regulates organ development and intracellular homeostasis,whose abnormality has been associated with tumors in the oral cavity (Ogi K et al., 2024). Flow cytometry and transwell indicate that treatment in mice showed that baicalin exhibited a dose-dependent inhibitory effect on the viability, proliferation and invasion of tongue cancer cells CAL27 accompanied by a reduction in the expression of Notch and JAG1 proteins as well as suppressed EMT, not accompanied by significant toxicity to normal human oral epithelial cells HOEC by MTT assay. In contrast, the Notch/JAG1 pathway activator VPA was able to reverse the above effects, confirming that baicalin exerts its antitumor effects through inhibition of the Notch/JAG1 pathway (Wang et al., 2023a). Knockdown of FTH1, a vital constituent of ferritin that is negatively correlated with OSCC differentiation, resulted in the upregulation of E-cadherin and downregulation of vimentin, snail, slug, MMP2, MMP9 in Cal27 and SCC25 cells, suggesting that FTH1 favors EMT, invasion and migration of OSCC cells. In contrast, baicalin was able to lead to upregulation of Fe^2+^, MDA, ROS and downregulation of GSH, reversing EMT induced by FTH1 overexpression and promoting ferroptosis (Wen Z et al., 2024). These above studies reflect a growing interest in the baicalin recently.

#### 4.1.3 Wogonin

Previous studies reported a direct inhibitory effect of wogonin concentration- and time-dependent on the proliferation of HN-6 cells, which was associated with cell arrest in the G0/G1 phase and apoptosis (Dong WX et al., 2017).

### 4.2 Esophageal cancer

EC, mainly consisting of two subtypes called esophageal squamous cell carcinoma (ESCC) and esophageal adenocarcinoma (EAC), is most common in East Asia and is becoming younger (Arnold M et al., 2020). Currently, the inhibitory effect of several flavonoids from *S. baicalensis* on EC has been demonstrated yet calls for more research (Table 2).

**TABLE 2:** Flavonoids of *Scutellaria baicalensis* in the treatment of EC.

Name	Dose	Subjects	Mechanism	Effect	Reference
Baicalein	1, 1.5, 2 mg/kg/d ip	OE19 cells xenograft NOG mice	PAK4↓	Inhibit proliferation	Liu et al. (2022)
	6.7, 20 μM	KYSE150 cells	HIF-1A↓PKM2↓	Inhibit proliferation, migration and invasion Promote apoptosis Induce cell cycle arrest at G1 phase Enhance sensitivity to chemotherapy	Guo D et al. (2022)
Baicalin	25, 50, 100, 200 μmol/L	ECA109 cells	Bad↑cIAP1↓	Inhibit proliferation Promote apoptosis	Liu SS et al. (2019)
Wogonin	10, 25, 50, 100, 150, 200 μM	KYSE150 cells	----	Inhibit proliferation Promote apoptosis Induce cell cycle arrest at G0/G1 phase	Huang WF et al. (2018)

#### 4.2.1 Baicalein

p21-activated kinase 4 (PAK4) is a serine threonine kinase, the levels of which correlate with the progression of a variety of cancers and could serve as a prognostic marker (Tang et al., 2023). Experiments *in vivo* showed that baicalein dose-dependently inhibited the growth of EC in mice with a decrease in PAK4 protein (Liu et al., 2023d).

Another study demonstrated that baicalein triggered G1 phase arrest and upregulation of L-phenyl propionamide, time- and dose-dependently inhibiting KYSE150 cell proliferation, migration and invasion. Furthermore, pretreatment of baicalein increased the sensitivity of tumor cells to 6Gy ray by down-regulating HIF-1A and PKM2, the key regulators of glycolysis. In conclusion, by interfering with the cellular glycolysis process, baicalein not only exerts a direct anti-EC effect, but also synergizes radiation therapy (Guo D et al., 2022).

#### 4.2.2 Baicalin

Time- and dose-dependent inhibition of ECA109 cell proliferation induced by baicalin is accompanied by upregulation of Bad, one of the major pro-apoptotic proteins of the Bcl-2 family, and downregulation of cIAP1, an apoptosis inhibitory protein belonging to the mitochondrial pathway in apoptosis, implying that baicalin can inhibit EC development through enhancing apoptosis (Liu SS et al., 2019).

#### 4.2.3 Wogonin

It was found that wogonin was able to block KYSE150 cells in the G0/G1 phase, directly inhibiting tumor cell proliferation and inducing apoptosis (Huang WF et al., 2018).

### 4.3 Gastric cancer

GC are malignant tumors with highly heterogeneous and invasive properties and young-onset has been on the rise in the last decade (Li Y et al., 2024). Early detection rates are low therefore often treated after complications in mid to late stages, which leads to dismal overall survival (Ren LF et al., 2024). The treatment of GC by *S. baicalensis* and its flavonoids *in vitro* and *in vivo* has been the focus of research in the last decade (Table 3).

**TABLE 3:** *Scutellaria baicalensis* and flavonoids in the treatment of GC.

Name	Dose	Subjects	Mechanism	Effect	Reference
*Scutellaria baicalensis*	20, 40, 80, 120, 160, 200 μg/mL	AGS and MGC-803 cells	p53↑p-Akt↓	Inhibit growth and proliferation	Cui et al. (2023)
Baicalein	30, 60, 120 μmol/L	SGC-7901 cells	Bax↑cleaved PARP↑Bcl-2↓	Inhibit proliferation, migration and invasion Promote apoptosis Induce cell cycle arrest at S phase	Mu J et al. (2016)
	10, 20, 40, 80, 120, 160, 200, 400 μM	SGC-7901 cells	MMP-2↓MMP-9↓	Inhibit proliferation, migration and invasion Promote apoptosis	Yan X et al. (2015)
	5, 15, 25 μmol/L	HGC-27cells	E-cadherin↑Vimentin↓	Inhibit proliferation, EMT Induce cell cycle arrest atG0/G1 phase	Duan YX et al. (2023)
	10, 50 μmol/L	MGC80-3, HGC-27, BGC-823 cells	GRP109A↑	Inhibit proliferation, migration and invasion	Hua WF et al. (2020)
	25, 50 μM	AGS cells	TGF-B↓Smad4↓N-cadherin↓vimentin↓ZEB1↓ZEB2↓	Inhibit migration and invasion	Chen F et al. (2014)
	15, 30, 60, 120 μM 48 h; 15, 50 mg/kg/d ig	AGS cells; AGS cells xenograft BALB/c mice	GRP78↑CHOP↑BTG3↑	Inhibit proliferation Promote apoptosis Induce cell cycle arrest at G0/G1 phase	Shen J et al. (2023)
	5, 15, 25 μmol/L	HGC-27 and SGC-7901 cells	E-cadherin↑cleaved Caspase-3↑Vimentin↓Snail↓MMP2↓MMP9↓Bcl-2↓p-PI3K↓p-AKT↓p-mTOR↓	Inhibit proliferation and migration Promote apoptosis	Qiao D et al. (2021)
	10, 20, 40, 60, 80 μM	AGS cells	PTEN↑p-Akt↓HIF-1α↓HK2↓LDHA↓PDK1↓	Inhibit proliferation Increase sensitivity to 5-FU	Chen F et al. (2015)
	2.5, 5, 10, 20, 40 μmol/L	SGC-7901 cells	----	Inhibit proliferation Promote apoptosis Increase sensitivity to oxaliplatin	Yang (2016)
	12.5, 25, 50, 100 μM	SGC-7901 cells	LC3 B↑p-IκBα↑p62↓p-mTOR↓p-Akt↓	Increase sensitivity to cisplatin	Li et al. (2020a)
Baicalin	40, 80, 120, 160 μmol/L	BGC-823 and MGC-803 cells	caspase-3↑caspase-9↑Bax↑Bcl-2↓	Inhibit proliferation Promote apoptosis	Wang et al. (2017b)
	10, 20, 40, 80, 160, 320 μmol/L	SGC-7901 cells	TLR8↑HIF-1α↑PDGF-β↑pten↑	Inhibit proliferation	Bai ZQ et al. (2017)
	50, 100, 200 μmol/L	SGC-7901 cells	Bax↑Bcl-2↓cyclinD1↓cyclinA1↓PI3K↓	Inhibit proliferation	Zheng XK et al. (2016)
	10, 20, 40, 80, 160, 320 μmol/L	MGC-803 and BGC-823 cells	FAS↑FASL↑TRAIL↑caspase-3↑caspase-8↑	Inhibit proliferation Promote apoptosis	Chen et al. (2015)
	40, 80, 120, 160 μmol/L	SGC-803 and BGC-823 cells	p53↑PTEN↑TIMP3↑MMP3↓	Inhibit migration	Wang et al. (2016a)
	100, 200, 400 μmol/L	SGC-7901 cells	PTGS2↑MDA↑p53↑GPX4↓SLC7A11↓	Inhibit viability	Yuan et al. (2023c)
	6.25, 12.5, 25, 50, 100 μM/mL	AGS cells	LDH↑GSDMD-N↑IL-18↑IL-1β↑Caspase-1↑ROS↑	Promote pyroptosis	Liu et al. (2024a)
	5, 10, 20, 40, 60, 80 μmol/L	SGC-7901 cells	PCNA↓	Inhibit proliferation Increase sensitivity to paclitaxel	Li et al. (2022a)
	30, 60, 90, 120, 150 ng/mL	AGS and SGC-7901 cells	TFR1↑NOX1↑COX2↑ROS↑FTH1↓FTL↓GPX4↓	Inhibit proliferation, migration and invasion	Yuan et al. (2023a)
Wogonin	20, 200 μmol/L	SGC-7901, BGC-823, MKN-45 cells	β-catenin↓C-myc↓Cyclin D1↓	Inhibit proliferation Promote apoptosis	Wang et al. (2016b)
	10, 20, 40, 80, 160 μM	MGC-803 cells	E-cadherin↑Vimentin↓ZEB1↓	Inhibit proliferation, migration and invasion via EMT	Dai JF et al. (2020)
	5, 10, 15, 20, 25, 30 μg/mL	SGC-7901 cells	LDH↓SDH↓ATP↓HIF-1α↓MCT4↓	Inhibit proliferation	Wang et al. (2019a)
	5, 10, 15, 20, 25, 30 μg/mL	SGC-7901 cells	HIF-1α↓MCT-4↓	Inhibit proliferation	Wang et al. (2019b)
	10, 50, 200 μM	BGC-823cells; BGC-823 cells xenograft zebrafish	p-JNK↑	Promote apoptosis	Hong ZP et al. (2018)

#### 4.3.1 Scutellaria baicalensis

Network pharmacological analysis predicted that the major components of *S. baicalensis* for the treatment of GC include wogonin, baicalein, acacetin, moslosooflavone, and oroxylin A, and that the major pathways are the PI3K-Akt, P53, and VEGF pathways. Subsequent experiments confirmed that *S. baicalensis* extract concentration-dependently inhibited the growth and migration of AGS and MGC-803 cells, accompanied by a decrease in the phosphorylation level of Akt proteins and an upregulation of the expression of p53 proteins. However, other mechanisms uncovered in this study still need to be further validated (Cui Y et al., 2023).

#### 4.3.2 Baicalein

Baicalein was reported to time- and dose-dependently inhibited proliferation and induced apoptosis in SGC-7901 cell, accompanied by S-phase arrest, which was consistent with the results of treatment *in vivo* (Mu J et al., 2016). Likewise, baicalein inhibit the proliferation and migration of GC cells SGC-7901 by down-regulating matrix metalloproteinase (MMP)-2 and −9 expression. While the p38 inhibitor SB203580 and activator chemical anisomycin were able to enhance and attenuate this anticancer effect, respectively, demonstrating that baicalein inhibits GC cell invasion and metastasis through the p38 signaling pathway (Yan X et al., 2015). Baicalein inhibited the proliferation, migration and invasion of MGC80-3, HGC-27 and BGC-823 cells, which was positively correlated with the expression of the nicotinic acid receptor GPR109A protein, a G-protein-coupled receptor with tumor-suppressive effect. Silencing this protein partially reversed the inhibitory effect of baicalein, suggesting that GRP109A is one of the targets of baicalein to inhibit the proliferation of GC (Hua et al., 2020). Transforming growth factor-B (TGF-B) is a multifunctional cytokine that regulates tumor cells (Rodrigues-Junior et al., 2024). Study have shown that baicalein reduces the expression of TGF-B, Smad4 and its downstream N-cadherin, vimentin, ZEB1, ZEB2, inhibiting AGS cell migration and invasion (Chen F et al., 2014). In addition, the role of baicalein in triggering cell cycle arrest and inhibiting EMT and proliferation in HGC-27 cells was also recognized (Duan YX et al., 2023). Endoplasmic reticulum stress (ERS), a universal cellular stress response, plays a very important role in the early adaptive survival and subsequent development of GC cells (Mommersteeg MC et al., 2022). Expression of B-cell translocation gene 3 (BTG3) regulates multiple life processes in GC cells by blocking the PI3K/AKT/mTOR pathway (Cheng YC et al., 2020). Baicalein inhibits cell proliferation and induced cellular G0/G1 cycle arrest and apoptosis in HGC-27 and AGS cells accompanied by an increase in ERS-associated GRP78, CHOP protein and BTG protein. Further treatment experiment by ERS blocker 4-PBA and PI3K inhibitor LY294002 reversely demonstrated that baicalein triggered ERS-induced apoptosis by blocking the PI3K/AKT pathway through activation of BTG3. Treatment in xenograft mice verified the above effect as well (Shen J et al., 2023). Focal adhesion kinase (FAK), which is often overexpressed in GC cells, is involved in the proliferation, survival and migration of tumor cells (Gao J et al., 2023). Baicalein dose-dependently upregulated E-cadherin, the cleaved Caspase-3 and downregulated the expression of Vimentin, Snail, MMP2, MMP9, Bcl-2, p-PI3K, p-AKT, and p-mTOR in HGC-27 and SGC-7901 cells, which appeared to inhibit tumor growth *in vivo* and *in vitro*. This indicates that baicalein inhibits cell migration and induces apoptosis by suppressing EMT. In addition, baicalein downregulates FAK expression, which inhibits the PI3K/AKT/mTOR signaling pathway and reduces cell viability, suggesting that FAK is one of the targets for baicalein to exert its therapeutic effects (Qiao D et al., 2021).

In addition to direct inhibitory effects, baicalein also enhances the effects of chemotherapeutic drugs at multiple targets. For example, rapid growth of malignant tumors tends to create a hypoxic microenvironment, which in turn can increase the resistance of tumor cells to chemotherapeutic drugs (Fu J et al., 2024). Hypoxia inducible factor-1α (HIF-1α) intensively participate in hypoxia-induced drug resistance in tumor cells, and its expression is inhibited by the oncogene PTEN (Shen G et al., 2022). A previous study found that baicalein concentration-dependently enhanced PTEN expression and attenuated HIF-1α, p-Akt, and glycolysis-associated enzymes hexokinase-2 (HK2), lactate dehydrogenase A (LDHA), pyruvate dehydrogenase kinase-1 (PDK1) expression, inhibited proliferation of AGS cell and reversed hypoxia-induced 5-FU resistance. This suggests that inhibition of glycolysis via the PTEN/Akt/HIF-1α pathway is one of the mechanisms underlying the anticancer effects of baicalein (Chen F et al., 2015). Baicalein was able to concentration-dependently increase the inhibitory effect of oxaliplatin on the proliferation of SGC-7901 cells and induce apoptosis (Yang CL, 2016). Similarly, baicalein would increase the sensitivity of cisplatin-resistant cells SGC-7901 cells to chemotherapeutic drugs accompanied by the upregulation of LC3 B, p-IκBα and the downregulation of p62, p-mTOR, and p-Akt as well as the regulation of Nrf2/Keap1 pathway (Li et al., 2020a).

#### 4.3.3 Baicalin

Time- and dose-dependent inhibition of BGC-823 and MGC-803 proliferation and induction of apoptosis by baicalin was previously reported (Wang et al., 2017b). The oncogene PTEN also inhibits the malignant behavior of tumor cells by negatively regulating the activation of the PI3K/Akt/mTOR pathway (Bao Y et al., 2024). Baicalin induces time- and dose-dependent inhibition of cell proliferation in SGC-7901 cells by upregulation of protein of TLR8, HIF-1α, PDGF-β and PTEN expression (Bai et al., 2017). Another study showed that baicalin (50–200 μmol/L 48 h) inhibited SGC-7901 cells with the upregulation of Bax and the downregulation of Bcl-2, cyclinD1, cyclinA1, and PI3K, suggesting that baicalin inhibits GC cell proliferation by blocking PI3K/Akt and its downstream pathway (Zheng et al., 2016). Factor associated suicide (FAS) and the corresponding factor associated suicide ligand (FASL) co-activate apoptosis (Li et al., 2017). TNF-related apoptosis-inducing ligand (TRAIL) has the potential to induce apoptosis in tumor cells too (Guerrache and Micheau, 2024). Experiment showed that baicalin time- and concentration-dependently inhibited MGC-803 and BGC-823 cell proliferation and induced apoptosis accompanied by upregulation of FAS, FASL, TRAIL, caspase3 and caspase8 expression. It is reasonable to speculate that the antitumor effects of baicalin may be related to apoptosis mediated by the death receptor pathway (Chen FQ et al., 2015). MMPs disrupt the histological barrier to accelerate tumor cell migration and, together with their inhibitors TIMPs, play a key role in tumor invasion and metastasis (Dibdiakova K et al., 2024). Migration of MGC-803 and SGC-823 cells inhibited by baicalin was accompanied by upregulation of p53, PTEN, and TIMP3 proteins and downregulation of MMP3 proteins (Wang et al., 2016a). Baicalin time- and concentration-dependently inhibited the activity of SGC-7901 cells without affecting normal cells GES-1, which could not be alleviated by apoptosis inhibitor Z-VAD-FMK and necrosis inhibitor Necrostatin-1. Following study revealed that the inhibition of cellular viability was accompanied by elevated levels of PTGS2, MDA, and p53, decreased levels of GPX and SLC7A11, and decreased activity of the antioxidant GSH. The above effects were attenuated by the addition of Fer-1, an ferroptosis inhibitor. It is evident that baicalin-induced p53-triggered downregulation of SLC7A11 is an important pathway of ferroptosis in GC cells (Yuan L. et al., 2023). It was found that baicalin upregulated LDH, GSDMD-N, IL-18, IL-1β, Caspase-1, NF-κB, IKKB, ROS, enhanced AGS cell pyrokinesis and dose-dependently reversed the effect of NLRP3 inhibitor MCC950 Sodium, suggesting the involvement of the NF-B/NLRP3 pathway (Liu J. et al., 2024).

Baicalin was able to inhibit the proliferation of SGC-7901 cells accompanied by a decrease in the proliferative protein PCNA, either alone or synergistically with paclitaxel, in a time- and concentration-dependent manner (Li LJ. et al., 2022). In addition, baicalin was able to concentration-dependently synergize with 5-FU to inhibit the growth, migration, and invasion of AGS and SGC-7901 cells accompanied by an increase in TFR1, NOX1, COX2, and ROS and a decrease in FTH1, FTL, and GPX4, which was reversed by the ferroptosis inhibitor Fer-1. Furthermore, baicalin does not kill normal epithelial cells GES-1, showing that ROS-mediated ferroptosis is one of the mechanisms by which baicalin is specifically anti-GC (Yuan J. et al., 2023).

#### 4.3.4 Wogonin

Earlier studies reported that wogonin (20–200 μmol/L 24–72 h) was able to inhibit the proliferation of SGC-7901, BGC-823, and MKN-45 cells in a time- and concentration-dependent manner. Further studies revealed that wogonin-induced apoptosis in SGC-7901 cells was accompanied by a decrease in the levels of β-catenin, C-myc, and Cyclin D1 proteins, suggesting that the therapeutic effect of wogonin on GC is associated with the inhibition of the Wnt/β-catenin signaling pathway (Wang et al., 2016a). In addition, wogonin dose-dependently inhibited the proliferation, erosion and migration of MGC-803 cells and suppressed the EMT process by up-regulating E-cadherin and down-regulating Vimentin, ZEB1 expression (Dai JF et al., 2020). Lactate acid generated during glycolysis not only provides energy to tumor cells, but also participates in the tumor microenvironment thereby promoting malignant behavior (Chen W. et al., 2024). Lactate dehydrogenase (LDH), as a key enzyme in glycolysis, promotes lactate acid production together with HIF-1α. Then, Monocarboxylate transporter-4 (MCT-4) transports lactate acid outside the cell and exacerbates the malignant behavior of tumor cells, whose blockade helps to reverse the immunosuppression of the tumor (Babl N et al., 2023). Study have confirmed that wogonin inhibited proliferation of SGC-7901 cells accompanied by a decrease in LDH and SDH viability and a decrease in ATP, HIF-1α, and MCT4 content (Wang et al., 2019a). Consistent with this, wogonin time- and dose-dependently inhibited proliferation of SGC-7901 cells and downregulated HIF-1α and MCT-4 expression and LDH, succinate dehydrogenase (SDH) activity and adenosine triphosphate (ATP) content, suggesting that wogonin counteracts GC by interfering with energy metabolism (Wang et al., 2019a).

Oxaliplatin in GC treatment often leads to neurological damage (Bennedsgaard K et al., 2020). Wogonin synergized with low dose oxaliplatin induced apoptosis in BGC-823 cells accompanied by an JNK (Thr183/Tyr185) increase in phosphorylation. And the synergistic treatment of the two concentration-dependently increased LC3II formation and decreased unc51-likekinase 1 (ULK1) (Ser555) expression. In addition, wogonin also potentiated the tumor inhibitory effect of oxaliplatin in a novel zebrafish model *in vivo*. These evidences together support the hypothesis that wogonin can enhance the anti-GC effect of oxaliplatin by inducing apoptosis and demonstrate the value of synergistic application of the two drugs to increase the effect and reduce side efforts (Hong ZP et al., 2018).

### 4.4 Colorectal cancer

CRC is the most prevalent tumor in digestive system and predominantly exists in the elderly population, whose occurrence is closely related to lifestyle (Xin J et al., 2024; Marino P et al., 2024). The current treatment of CRC mainly includes surgery, radiotherapy, chemotherapy, and immune therapy (Shebbo S et al., 2024). Hopefully, the effects of *S. baicalensis* and flavonoids have been revealed (Table 4).

**TABLE 4:** *Scutellaria baicalensis* and its flavonoids in the treatment of CRC.

Name	Dose	Subjects	Mechanism	Effect	Reference
*Scutellaria baicalensis*	100 mg/mL; 4 g/Kg/d ig	HT29, MC38, H630-R1, RKO-R10,CCD841 cells; MC38 cells xenograft C57BL/6mice	TS↓E2F1↓RB↓CDK4↓CDK6↓cyclin D1↓	Inhibit proliferation Increase sensitivity to 5-Fu and capecitabine	Liu et al. (2023a)
Baicalein	0, 5, 10, 20, 40, 80 μg/mL	HT-29 cells	STAT3↓NF-κB↓ p53↑	Inhibit proliferation and migration	Zhao XY et al. (2015)
	20, 40, 40 μmol/L	HCT-29 cells	12-LOX↓		Xu L et al. (2020)
	20, 40, 80 μmol/L	SW480 cells	cleaved-caspase3↑cleaved-PARP↑p-MET/MET↓p-Akt/Akt↓p-H3/H3↓	Inhibit proliferation Promote apoptosis	Xu JL et al. (2022)
	10, 20, 40, 80, 160 μM	HT-29, HCT-116, SW480, SW620 cells	LC3-Ⅱ↑caspase-3↑BIRC3↑	Inhibit viability, autophagy Promote apoptosis	Phan T et al. (2020)
	10, 20, 40 μM	HCT116 cells	DEPP↑Gadd45a↑cleaved caspase-3↑cleaved caspase-9↑p-JNK↑p-ERK↑p-p38↑	Inhibit proliferation Promote apoptosis	Su MQ et al. (2018)
	20, 40, 60, 80, 100, 120 mol/L; 4.5 g/kg ig	HT29, DLD1 cells; Sprague-Dawley mouse	p53↑p21↑E-cadherin↑Snail↓Twist1↓Vimentin↓	Inhibit EMT, proliferation, migration and invasion	Zeng Q et al. (2020)
	50, 100, 200 μmol/L	HT-29 cells	p-YAP↑p-LATS↑p-Ser↑	Inhibit proliferation	Meng XC et al. (2022)
Baicalin	50, 100, 200 mg/kg ig bid	HCT-116 cells xenograft BALB/C mice		Inhibit proliferation Promote apoptosis Induce cell cycle arrest at G2/M phase	Xu ZZ et al. (2017)
	4, 8, 16, 32, 64, 128 μmol/L; 40 mg/kg/d ip	SW620, NCM460 cells; CRC mice induced by AOM/DSS	Caspase-3↑Caspase-9↑SUFU↑IL-1β↓IL-6↓TNF-α↓SHH↓SMO↓Gli1↓	Inhibit proliferation	Lin H et al. (2023)
	50, 100, 150, 200 μg/mL; 50 mg/kg/d ip	SW480, HCT116, HT26, CT26 cells; CT26 cells xenograft BALB/c mice	miR-139-3p↑CDK16↓	Inhibit proliferation Induce cell cycle arrest at S phase	Cai et al. (2023a)
	5, 10, 20, 40, 80 μg/mL; 20, 40 mg/kg/d ip	HCT-116 and CT26 cells; CT26 cells xenograft BALB/c mice	cleaved caspase3↑ROS↑TIMP-2↑MMP-2↓MMP-9↓TLR-4↓NF-κB p65↓p-IκBα↓PD-L1↓	Inhibit proliferation, migration and invasion Promote apoptosis	Song L et al. (2022)
	200 mg/kg/d ig	MC38 cells; MC38 cells xenograft C57BL/6J mice	E-cadherin↑Occludin↑Vimentin↓N-cadherin↓	Inhibit EMT, metastasis Improve the gut microbiota	Wei J et al. (2023)
Wogonin	10, 20, 40 μM; 20, 40, 80 mg/Kg/qod 20 d	HCT116, A2780, HT29 cells; A2780 or HT29 cells xenograft BALB/c mice	TIGAR↑PGM↓HK2↓GLUT1↓PDHK1↓LDHA↓	Inhibit cell viability	Zhao Y et al. (2018)
	20, 40, 80, 160 μg/mL	SW620, SW480, HT29, HCT116, LOVO cells	BAX↑Bcl-2↓	Inhibit EMT and proliferation Promote apoptosis	Mao HY et al. (2021)
	6.25, 12.5, 25, 50 μmol/L	SW480 cells	BAX↑CTNNB1↓GSK3B↓BIRC5↓	Promote apoptosis Induce cell cycle arrest at G1	Li et al. (2020b)
	0.5, 1, 2, 4μM; 2 μM/d/qod ip	SW480, HCT116 cells; SW480 cells xenograft BALB/c mice	E-cadherin↑vimentin↓ZEB2↓N-cadherin↓SMAD3 ↓YAP1↓AXL↓CYR61↓CTGF↓IRF3↓	Inhibit survival, EMT, migration and invasion	You W et al. (2022)
	25, 50 μM	LOVO and LOVO/DX cells	----	Inhibit migration Promote apoptosis	Radajewska A et al. (2023)
Scutellaria flavone Ⅰ	80 μmol/L	LOVO cells	E-cadherin↑miR-378↑Vimentin↓N-cadherin↓	Inhibit EMT, migration and invasion	Zhang et al. (2021b)
Scutellarin	20, 40, 80, 120, 160, 200, 240, 280, 300 μg/mL	HCT-116 cells	caspase-3↑caspase-9↑Bax↑MST1↑LATS1↑Bel-2↓p-YAP1↑YAP1↓TAZ↓c-Myc↓	Inhibit survival and migration Promote apoptosis	Yang H et al. (2023)
	40, 80, 160 g/mL; 25, 50, 100 mg/kg/d ip	HT29-CSC cells; HT29 cells xenograft BALB/c mice	Gli1↓Ptch1↓CD133↓Lgr5↓c-Myc↓Ki-67↓CK20↓Nanog↓	Inhibit proliferation and differentiation	Lei N et al. (2020)
	40, 80, 120, 160, 200, 240, 280 μmol/L	HCT-116 cells	cleaved caspase-3↑p53↑p-ERK1/2↑p62↓c-Met↓Akt↓	Promote apoptosis Increase sensitivity to oxaliplatin	Yang HJ et al. (2022)

#### 4.4.1 Scutellaria baicalensis

*Scutellaria baicalensis* concentration-dependently inhibited the proliferation of CRC cell lines HT29, MC38, chemotherapy-resistant cells H630-R1 and RKO-R10, and normal cells CCD841 *in vitro* directly. Further studies revealed that *S. baicalensis* treatment induced sub-G0 phase arrest and downregulated the expression of TS, E2F1, RB, CDK4, CDK6, and cyclin D1, exposing that the inhibition of the CDK-RB pathway may be one of the mechanisms of CRC suppression. Moreover, *S. baicalensis* enhanced the inhibition of 5-FU in drug-resistant H630-R1 and RKO-R10 cells, accompanied by the downregulation of TS and ITC. Animal studies showed that *S. baicalensis* exhibited synergistic effects with 5-FU or capecitabine and did not show significant toxicity. Notably, oral or intraperitoneal injection of baicalin did not have a significant therapeutic effect in animal studies and deserves further research on its dosage (Liu et al., 2023a).

#### 4.4.2 Baicalein

Earlier studies found that baicalein dose-dependently inhibited the proliferation and migration of HT-29 cells, accompanied by elevated p53 levels and decreased STAT3, NF-κB, suggesting that this anticancer effect may be achieved by promoting TIGAR gene expression and STAT3 pathway (Zhao et al., 2015). Precious study has confirmed that 12-lipoxygenase (12-LOX), a key enzyme in the arachidonic acid metabolic pathway, has sequentially increased expression in normal, adenoma, and CRC tissues, and is able to promote CRC invasion and metastasis (Li S et al., 2013). Baicalein has shown the time- and dose-dependent reduction of 12-LOX mRNA expression in HT-29 cells (Xu L et al., 2020). Aberrant activation of mesenchymal epithelial transition factor (MET) and phosphatidylinositol 3-kinase (PI3K)/protein kinase B (Akt) has been shown to correlate with a wide range of malignant behaviors in CRC (Leiphrakpam and Are, 2024). Baicalein was reported to reduce the levels of p-MET/MET, p-Akt/Akt and p-H3/H3, increased the levels of apoptosis-associated proteins cleaved-caspase3 and cleaved-PARP, concentration-dependently inhibiting the MET/Akt signaling pathway and thus the SW480 proliferation and promoting apoptosis. In addition, baicalein treatment induced a decrease in ROS generation and (superoxide dismutase, SOD), (catalase, CAT) activity compared to the control group (Xu JL et al., 2022). Baicalein dose-dependently decreased the viability of HT-29, HCT-116, SW480, and SW620 cells, which could be enhanced by the autophagy inhibitor chloroquine (CQ). Analysis showed increased expression of LC3-II, caspase-3, and BIRC3, suggesting that the therapeutic effects of baicalein correlate with inhibited autophagy and enhanced apoptosis (Phan T et al., 2020). Decidual protein induced by progesterone (DEPP) always enhances ROS-induced tumor cell death (Salcher S et al., 2014). Growth arrest and DNA damage-inducible 45a (Gadd45a) is an important cell cycle regulator counteracting tumor growth (Palomer X et al., 2024). Baicalein could inhibit proliferation and induces apoptosis of HCT116 cells, accompanied by upregulation of DEPP, Gadd45a, cleaved caspase-3, cleaved caspase-9, p-JNK, p-ERK, and p-p38. Subsequent knockdown of DEPP and Gadd45a attenuated the effects of baicalein. In conclusion, baicalein induces apoptosis in CRC cells through the JNK/ERK/p38 signaling pathway (Su MQ et al., 2018). Baicalein time- and dose-dependently inhibited viability, migration and invasion of HT29 and DLD1 cells, accompanied by an increase in p53, p21, E-cadherin and a decrease in Snail, Twist1, Vimentin. This suggests that baicalein inhibits EMT in CRC cells by decreasing Snail activity (Zeng Q et al., 2020).

In addition, baicalein has been proved to dose-dependently potentiate the inhibitory effect of irinotecan on proliferation of HT-29 cells accompanied by the inhibitory effect of Yes-related protein (YAP), large-tumor suppressor kinase 1 (LATS1), and phosphorylation of Src, which plays an important role as a non-receptor protein complex kinase in EMT of CRC (Meng XC et al., 2022; Sadri F et al., 2023).

#### 4.4.3 Baicalin

DNA-mismatch repair (MMR) maintains genetic stability by correcting mismatched DNA bases, whereas defective DNA mismatch repair (dMMR) induces DNA mis replication and microsatellite instability (MSI) instability leading to an increased CRC pathogenesis increased risk (Moreau M et al., 2024). In dMMR nude mice, baicalin increases the expression of MMR genes hmlH1 and hMSH2, causing G2/M phase arrest and apoptosis in HCT-116 cells, thereby inhibiting tumor growth (Xu ZZ et al., 2017). Hedgehog signaling pathway plays an important role in the inflammatory cancerous transformation of CRC, consisting of activation of smoothened (SMO) by elevated sonic hedgehog (SHH), alleviation of serine/threonine kinase (SUFU) activation, translocation of Glioma (Gli1) proteins, and ultimately cellular hyperproliferation (Wu H et al., 2023). Baicalin was able to time- and dose-dependently inhibit proliferation of SW620 cells, which was accompanied by an increase in caspase-3, caspase-9, SUFU activity and a decrease in IL-1β, IL-6, TNF-α, SHH, SMO, and Gli1 levels. Experiments in CRC mice have also confirmed the effect of baicalin on the Hedgehog pathway inhibition. However, prolonged and high doses of baicalin also inhibited normal colonic epithelial NCM460 cells, warning that its overuse should be guarded against in the clinic (Lin H et al., 2023). Cyclin-dependent kinase 16 (CDK16) regulates cell differentiation in physiological state while favoring tumor development in pathological state, and can be used as a marker for the prognostic situation of CRC (Guan L et al., 2022). Baicalin inhibited the prognostic status of CRC *in vitro* by increasing the miR-139-3p and decreasing the CDK16 levels, resulting in S-phase arrest and cell viability inhibition in SW480, HCT-116, and CT26 cells, which can be reversed by miR-139-3p silencing and CDK16 overexpression. Experiments *in vivo* also support the conclusion that baicalin treats CRC by modulating the miR-139-3p/CDK16 axis (Cai R. et al., 2023). Baicalin exerts anti-proliferative, anti-migratory, anti-erosive and pro-apoptotic effects in HCT-116 and CT26 cells and did not lead to pathological changes in animals. Meanwhile, the increase in PD-L1 levels and decrease in TLR-4, NF-κB p65, and p-IκBα levels imply that baicalin functions by improving immunity and inhibiting the TLR-4/NF-κB pathway (Song L et al., 2022). Animal experiments revealed that a high-fat diet led to enhanced CRC invasiveness by elevating E-cadherin and Occludin mRNA levels and decreasing Vimentin and N-cadherin mRNA levels. Treatment with baicalin was able to reverse the resulting CRC live metastasis by inhibiting EMT in animals compared to controls, a process that was accompanied by an improvement in the composition of the gut microbiota (Wei J et al., 2023).

#### 4.4.4 Wogonin

It was shown that low doses of wogonin dose-dependently inhibited the survival of HCT116 and HepG2 cells, which express wp-p53, by up-regulating TIGAR and down-regulating PGM, HK2, GLUT1, PDHK1, and LDHA. These effects were also observed in ovarian cancer A2780 cells in xenograft mice, while absent in p53-deficient HCT116 *in vitro* and HT-29 cells *in vivo*. Further studies revealed that wogonin inhibited the interaction of p53 with its degradation factor MDM2. Thus, inhibition of glycolysis due to p53 stabilization is involved in the anti-tumor effects of wogonin (Zhao Y et al., 2018). Biliverdin reductase A (BLVRA), a soluble NADPH-dependent enzyme, functions by maintaining intracellular redox reactions and its elevation favors CRC growth (Mao H et al., 2020). The expression of BLVRA in the CRC cell lines SW620, SW480, HT29, HCT116, and LOVO were all significantly higher than in normal intestinal epithelial FHC cells, which is consistent with the report. Further experiment revealed that wogonin had a time- and concentration-dependent inhibitory effect on the proliferation of HT29 and SW620 cells, which was accompanied by increased levels of apoptosis, EMT inhibition and decreased BLVRA expression (Mao HY et al., 2021). Wogonin was shown to dose-dependently inhibit proliferation of SW480 cells and induce the cell cycle arrest in G1 phase, accompanied by the upregulation of BAX and the downregulation of CTNNB1, GSK3B, and BIRC5, demonstrating that wogonin also counteracts CRC by inhibiting the Wnt/β-catenin pathway (Li et al., 2020b). Interferon regulatory factor 3 (IRF3), which often suggests a poor prognosis for CRC patients, is an agonist of YAP1 and a target for tumor therapy (Chen YJ et al., 2021). Wogonin inhibited survival, migration and invasion of SW480 and HCT116 cells accompanied by upregulation of E-cadherin and downregulation of vimentin, ZEB2, N-cadherin, SMAD3 as well as YAP1, AXL, CYR61, CTGF, and IRF3, which were reversed by YAP1 overexpression. Animal experiments were consistent with the above results. It is evident that wogonin inhibits the EMT process in CRC by regulating the IRF3-mediated Hippo pathway (You W et al., 2022).

Meanwhile, cellular experiments have shown that adding wogonin would synergize with irinotecan to promote apoptosis and inhibit migration of drug-sensitive LOVO and doxorubicin-resistant LOVO/DX cells directly. However, their synergistic effects in tumor-bearing animals are still unknown (Radajewska A et al., 2023).

#### 4.4.5 Others

In addition to the above, other flavonoids of *S. baicalensis* also have therapeutic effects on CRC. For example, Scutellaria flavone Ⅰ inhibited EMT by up-regulating E-cadherin and down-regulating N-cadherin, and Vimentin accompanied by miR-378 elevation, inhibiting migration and invasion of LOVO cells (Zhang et al., 2021b). Transcriptional co-activator with PDZ-binding motif (TAZ) is an important target of the Hippo pathway and contributes to CRC angiogenesis (Shen Y. et al., 2021). Scutellarin concentration-dependently inhibited survival and migration and induced apoptosis of HCT-116 cells accompanied by upregulation of LATS1, MST1, p-YAP and downregulation of YAP1, TAZ, c-Myc, suggesting that scutellarin may induce apoptosis through activation of Hippo-YAP/TAZ pathway in CRC cells (Yang H et al., 2023). The tumor stem cell markers Lgr5 and Nanog are important reference for CRC development (Ahmed EM et al., 2023; Vasefifar P et al., 2022). Scutellarin concentration-dependently inhibited the growth and transformation of tumor stem cells HT-29CSC *in vitro* accompanied by a decrease in the expression of Lgr5, CK20. Treatment on animals showed that scutellarin reduced the expression levels of Gli1, Ptch1, CD133, Lgr5, c-Myc, Ki-67, CK20, and Nanog. It can be seen that scutellarin interferes with CRC stem cell differentiation *in vitro* and *in vivo* by inhibiting the hedgehog pathway (Lei N et al., 2020).

More than direct therapeutic effects, scutellarin was also shown to dose-dependently enhance the effects of oxaliplatin in promoting apoptosis in HCT-116 cells accompanied by the upregulation of p53, p-ERK1/2 and the decrease of c-Met, Akt, which may be associated with the activation of ERK/p53 pathway and inhibition of c-Met, Akt./p53 pathway activation and c-Met/Akt pathway. This suggests that the mitochondrial pathway is also involved in the treatment of colorectal cancer with scutellarin (Yang HJ et al., 2022).

### 4.5 Hepatocellular cancer

HCC percentages 75%–85% of tumors in liver, which mostly develops from chronic liver disease and is widely distributed in East Asia and North Africa. Currently, its treatment mainly includes multiple kinase inhibitors (MKIs), such as sorafenib and regorafenib, ablation, surgery and immunotherapy (Chen W et al., 2024). In recent years, the therapeutic role of *S. baicalensis* in HCC has been gradually revealed through the intervention of ferroptosis, apoptosis, EMT and other mechanisms (Table 5). A recent meta-analysis suggested the efficacy and safety of *S. baicalensis* and its flavonoids in HCC treatment (Ma et al., 2023a).

**TABLE 5:** *Scutellaria baicalensis* and its flavonoids in the treatment of HCC.

Name	Dose	Subjects	Mechanism	Effect	Reference
*Scutellaria baicalensis*	0.65, 1.25, 2.5, 5, 10 μM	HepG2 and Huh7 cells	ROS↑JUN↑RELA↑AKT1↑	Inhibit viability	Cai et al. (2023b)
	3.15, 6.3, 12.5, 25, 25, 50 mg/mL; 140 g/d ig	SMMC-7721, HepG2, Huh7 cells; HepG2 or Huh7 cells xenograft BALB/c mice	IREB2↑ACSL4↑GPX4↓SLC7A11↓	Inhibit proliferation Promote ferroptosis	Li et al. (2022b)
	15.625, 31.25, 62.5, 125, 250, 500, 1000 μg/mL	SK-Hp-1 cells	cleaved caspase-3↑caspase-7↑caspase-9↑PARP↑p53↑Bax↑E-Cadherin↑claudin↑HSP60↑Bcl-2↓CDK2↓CDK4↓CDK6↓cyclin D↓cyclin E↓N-Cadherin↓vimentin↓HSP90β↓HSP70↓	Inhibit EMT Promote apoptosis Induce cell cycle arrest at G1/S	Wu et al. (2024b)
Baicalein	1, 2, 5, 10, 20, 50, 100, 200, 500 mol/L	SMMC-7721 cells	P-ERK1/2↓CyclinD1↓P-GSK-3β↓P-AKT↓	Inhibit proliferation	Wang et al. (2017a)
	1, 10, 40, 80, 160μM; 80 mg/kg/d ip	HMCC-97H and SMCC-7721 cells	miR-3178↑HDAC10↓	Inhibit proliferation Promote apoptosis	Qi J et al. (2023)
	2.5, 5, 10, 20, 40 μM	SMMC-7721 and HepG2 cells	PD-L1↓	Inhibit proliferation Promote immune response	Ke M et al. (2019)
	12.5, 25, 50, 100M; 10 mg/kg/d ip	SMMC-7721, Hep3B, HCCLM3, HepG2 cells; SMMC-7721 cells xenograft BALB/c mice	----	Inhibit proliferation and migration	Yu X et al. (2018)
	1, 2, 5, 10, 20, 50, 100, 200, 300 μM	SMMC-7721 cells	Bax↑Bcl-2↓Akt↓ERK1/2↓GSK-3β↓	Promote apoptosis Induce cell cycle arrest at G0/G1	He K et al. (2018)
	10, 20, 40, 80, 160 μg/mL	Bel7402 cells	Bax↑Bcl-2↓	Promote apoptosis Increase sensitivity to 5-FU and epirubicin	Li et al. (2018a)
	31.25, 62.5, 125, 500 μg/mL	HepG2 cells	Bax↑ beclin 1↑TGFβ1↓ATG-7↑	Promote apoptosis Increase sensitivity to epirubicin	Al-Ashmawy GM et al. (2024)
Baicalin	25, 50, 100 μg/mL	HepG2 cells	Bax↑ Bcl-2↓	Promote apoptosis	Xie YH et al. (2023)
	50, 100, 200, 300 μmol/L	HepG2 cells	Fe^2+^↑ ROS↑; SLC7A11↓ GPX4↓ GSH↓ p-PI3K/PI3K↓ p-Akt/Akt↓ p-FoxO3a/FoxO3a↓	Inhibit proliferation Promote ferroptosis	Zhou et al. (2024)
	2.5, 5, 7.5, 10, 12.5 μg/mL 12, 24, 48 h	HepG2 cells	p-MET↓p-EGFR↓	Inhibit EMT and proliferation Promote apoptosis Induce cell cycle arrest at G1 phase	Hu ZP et al. (2023)
	10, 20, 40, 60, 80, 120 μM	Hep3B and MHCC-97H cells; NC-MHCC-97H or ROCK1-UP-MHCC-97H cells xenograft BALB/c mice	Bax↑GSK-3β↑p-β-catenin↑p-GSK-3β↓p-catenin↓Cyclin D1↓VEGFA↓MMP-9↓Bcl-2↓	Inhibit proliferation, migration and invasion Promote apoptosis Induce cell cycle arrest at G0/G1 phase	Sun et al. (2023a)
Wogonin	37.5, 75, 150 μmol/L	HepG2 and LO2 cells	CDK1↓SRC↓	Inhibit proliferation and migration Promote apoptosis	Yang et al. (2024c)
	3.125, 6.25, 12.5, 25, 50, 100, 200 μM	SMMC-7721 and HCCLM3 cells	p21↑p-MOB1↑p-LATS↑Claspin↓CTGF↓CYR61↓	Promote apoptosis Induce cell cycle arrest atG2/M	Wu et al. (2023)
	20, 40, 80μM; 50 mg/kg/d ip	Huh7 cells; Huh7 cells xenograft BALB/c mice	miR-27b-5p↑YWHAZ↓	Inhibit proliferation Promote apoptosis Induce cell cycle arrest at G1/S phase	(Ma et al., 2023)
	50, 100, 200, 400, 800μM; 25, 50 mg/kg/d ip	MHCC97L, HepG2, LO2 cells; MHCC97L cells xenograft BALB/c mice	Cyclin D1↓	Inhibit proliferation Induce cell cycle arrest at G1 phase	Hong M et al. (2020)
Wogonoside	1, 2, 4, 8, 16, 32, 64, 128, 256, 512μM, 1mM, 2 mM	Bel7402 cells	Bax↑ Bcl-2↓	Inhibit proliferation Promote apoptosis Induce cell cycle arrest at G2/M	Li Y et al. (2015)
Oroxylin-A	12.5, 25, 50 μM 24h; 200 mg/kg/qod ig	SMMC-7721, HepG2, MHCC-97H cells; SMMC-7721 cells xenograft BALB/c mice	E-cadherin↑N-cadherin↓Vimentin↓Twist↓	Inhibit proliferation, EMT, and migration	Huo TX et al. (2022)
	2.5, 5, 10, 20, 40, 80 μM	HepG2 cells xenograft BALB/c mice	wt-p53↑p-MDM2↓p-SIRT↓	Inhibit viability	Yao et al. (2022b)
	10 μM 24h; 300 mg/kg/qod ig	HepG2 cells xenograft NOD/SCID mice	SIRT3↓FOXO3↓BNIP3↓PINK1↓PRKN↓	Inhibit autophagy Increase sensitivity to adriamycin	Yao et al. (2022a)
	6, 8, 10, 15, 20, 25 μM; 300 mg/kg/d ig	HepG2 and SMMC-7721 cells; HepG2 cells xenograft NOD/SCID mice	ALB↑HNF-4α↑PKM1↑PTB↓AFP↓PKM2↓	Inhibit proliferation Promote differentiation Induce cell cycle arrest at G2/M phase	Wei L et al. (2017)
	10 μM; 300 mg/kg/d ig	HepG2, SMMC-7721, H22, THP-1, HEK293T cells; H22 cells xenograft mice	MHC-Ⅱ↑CD-206↓	Promote apoptosis and immune response	Wang et al. (2023e)
	12 μM 48h; 300 mg/kg/qod ig	HepG2, Huh7, SMMC-7721 cells; HepG2 or SMMC-7721 cells xenograft mice	FIS1↑p-DRP1-s616↑OPA1↓p-DRP1-s637↓GLUT1↓SIRT1↓PDK2↓PARL1↓MFN1↓OPA1↓YME1L1↓PGC-1α↓	Promote apoptosis	Guo Y et al. (2023)

#### 4.5.1 Scutellaria baicalensis

Network pharmacological analysis suggested that JUN, RELA, and AKT1 might be the key targets for *S. baicalensis* to exert therapeutic effects on HCC. Subsequent experiments demonstrated that wogonin and baicalein could concentration-dependently inhibit HepG2 and Huh7 cell viability accompanied by elevated levels of ROS and mRNA expression of JUN, RELA, and AKT1, respectively, which provided a reference for future studies (Cai X. et al., 2023). Iron-responsive element binding protein 2 (IREB2), glutathione peroxidase 4 (GPX4), synthetase long chain family member 4 (SLC7A11) are important factors regulating ferroptosis (Fan H et al., 2022; Zhang W. et al, 2024; Koppula P et al., 2021). It was found that Scutellaria Barbata extract dose-dependently inhibited the growth of SMMC-7721, HepG2, and Huh7 cells compared to the blank control group, which was accompanied by a decrease in the ferroptosis inducers GPX4 and SLC7A11 proteins, and a decrease in the ferroptosis inhibitors IREB2 and ACSL4 proteins. In addition, animal experiments also demonstrated the inhibitory effect of *S. baicalensis* on tumor growth accompanied by a decrease in Ki-67 and SLC7A11 protein levels in xenograft mice. It can be seen that the induction of ferroptosis in HCC cells via iron perioxidation and lipid ROS metabolism is one of the mechanisms by which *S. baicalensis* exerts its therapeutic effects (Li Y. et al., 2022). Heat shock protein 90 (HSP90), a class of cellular chaperone proteins, widely affects the survival and proliferation of tumor cells (Tausif YM et al., 2024). *Scutellaria baicalensis* extract dose-dependently induced G1/S phase arrest and apoptosis in SK-Hp-1 cells and reversed the aberrant expression of EMT-related proteins without damaging normal hepatocytes. Moreover, the extract also enhanced the anticancer effect due to the inhibition of HSP90β, which has been shown to be associated with poor prognosis in advanced HCC (Wu TH. et al., 2024).

#### 4.5.2 Baicalein

Glycogen synthase kinase 3-β (GSK-3β) is closely related to the development of various tumors (Wang J. et al., 2024; Fukuda J et al., 2024). It was demonstrated that baicalein synergized with the PI3K pathway inhibitor LY294002 to inhibit the proliferation of SMMC-7721 cells without affecting apoptosis, a process that was associated with reduced expression of P-ERK1/2, CyclinD1, P-GSK-3β, and P-AKT (Wang et al., 2017a). MiR-3178, which can inhibit tumor cells by affecting EMT, decreased in HCC tissues and Bel-7402, Bel-7404, SMMC-7721, MHCC-97H, HepG2, Hep3B, and Huh7 cell lines compared to normal liver tissue L-O2 cells. Further intervention revealed that baicalein time- and dose-dependently inhibited proliferation and promoted apoptosis of HMCC-97H and SMCC-7721 cells, similar to sorafenib, accompanied by elevated miR-3178 and decreased HDAC10. Overexpression of miR-3178 decreased HDAC10 expression and thus HCC cell viability. Animal experiments showed the same therapeutic effect (Qi J et al., 2023). Increasing evidence suggests that upregulation of immune checkpoints, such as the programmed cell death-ligand 1 (PD-L1)/programmed cell death protein 1 (PD1) pathway, is an important way for tumor cells to achieve immune evasion (Hayashi H et al., 2024). Animal experiments revealed that baicalein and baicalin inhibited the growth of HCC accompanied by decreased PD-L1 expression in mice. Further studies revealed that baicalein and baicalin significantly inhibited IFN-γ-induced cellular PD-L1 upregulation thereby increasing T-cell-mediated tumor-killing activity in addition to dose-dependently and directly inhibiting proliferation of SMMC-7721 and HepG2 cell. In addition, both *in vivo* and *ex vivo* experiments demonstrated that inhibition of PD-L1 is associated with inhibition of STAT-3 phosphorylation (Ke M et al., 2019). A significant portion of HCC initiation and recurrencies derived by tumor initiating stem cell-like cells (TICs), whose marker CD133 expression level is negatively correlated with the final outcome of HCC patients (Wu J. et al., 2024). And it is reasonable to believe that TICs are closely related to HCC chemoresistance (Huang H et al., 2023). NF-κB interacting LncRNA (NKILA), an important regulator in tumor development, was found to be downregulated in SMMC-7721, Hep3B, HCCLM3, and HepG2 cells compared to normal hepatocytes and interacted closely with baicalein: overexpression of NKILA increased the expression of the inhibitory effects of baicalein on the proliferation and migration of SMMC-7721 and HepG2 and its knockdown reversed these effects, which was also verified in animal experiments (10 mg/kg/d 28 d). Further studies revealed that NKILA enhances the inhibitory effect of baicalein on NF-κB transcriptional activity, and the NF-κB inhibitor JSH-23 disrupts this synergy, implying that the combination of these is a promising therapeutic strategy (Yu X et al., 2018).

Furthermore, baicalein has been shown to synergize with the PI3K inhibitor LY294002 to induce G0/G1 phase arrest and apoptosis in SMMC-7721 cells (He K et al., 2018). Baicalein also induced apoptosis in SMMC-7721 cells by reversing the resistance of Bel7402 cells to chemotherapeutic drug (5-FU and epirubicin) and induced apoptosis (Li J. et al., 2018). Another study showed that baicalein enhances the toxicity of epirubicin on HepG2 cells by up-regulating the activation of autophagy by beclin 1 and ATG-7 (Al-Ashmawy et al., 2024). Addition of baicalein to antagonize the resistance of HCC cells to chemotherapeutic drugs is a feasible approach.

#### 4.5.3 Baicalin

Baicalin was shown to dose-dependently upregulate Bax and downregulate Bcl-2 protein expression to induce apoptosis in HepG2 cells directly (Xie YH et al., 2023). A recent study found that baicalin time- and dose-dependently inhibited proliferation of HepG2 cells, accompanied by a decrease in SLC7A11, GPX4, GSH, p-PI3K/PI3K, p-Akt/Akt, p-FoxO3a/FoxO3a levels and an increase in Fe^2+^, ROS. Fer-1 reversed these effects, revealing that the anti-HCC effects of baicalin is associated with inhibition of the ROS-mediated PI3K/Akt/FoxO3a pathway and ferroptosis (Zhou JQ et al., 2024). Epidermal growth factor receptor (EGFR) regulation of target genes and mesenchymal MET are closely associated with the malignant phenotype of cancer cells (Bhushan B et al., 2019). Baicalin was able to inhibit proliferation of HepG2 cells alone or in concert with the EGFR inhibitor gefitinib and the MET inhibitor crizotinib, triggering G1-phase cell arrest and induction of apoptosis accompanied by a decrease in p-MET, p-EGFR protein expression (Hu ZP et al., 2023). ROCK1 promotes migration and invasion of multiple tumors including HCC (Dong S et al., 2023). Baicalin induced G0/G1 phase arrest and apoptosis in Hep3B and MHCC-97H cells time- and dose-dependently, thereby inhibiting the proliferation, migration and invasion of HCC cells. Meanwhile, the expression of Bax, GSK-3β, and p-β-catenin was upregulated while that of ROCK1, p-GSK-3β, β-catenin, C-myc, cyclin D1, VEGFA, MMP-9, and Bcl-2 was downregulated, which was consistent with the alterations *in vivo* in mice. This suggests that baicalin may inhibit proliferation and metastasis of HCC through the ROCK1/GSK-3β/β-catenin pathway (Sun et al., 2023a).

#### 4.5.4 Wogonin

Network pharmacological analysis showed that wogonin has 113 intersecting targets with HCC, which is mainly focused on the PI3K/AKT signaling pathway. Subsequent experiments demonstrated that wogonin inhibited proliferation and migration and promoted apoptosis of HepG2 cells by down-regulating CDK1 and SRC expression, which was not significant in normal LO2 cells (Yang et al., 2024c). Similarly, wogonin concentration-dependently induced G2/M cell cycle arrest and apoptosis in SMMC-7721 and HCCLM3 cells accompanied by upregulation of p21, p-MOB1, p-LATS and downregulation of Claspin, CTGF, and CYR61, which could be reversed by YAP or TAZ overexpression. It is evident that the pro-apoptotic effect of wogonin in HCC involves activation of MOB1/LATS and inhibition of YAP/TAZ in the Hippo pathway (Wu K et al., 2022). Wogonin inhibited the proliferation of Huh7 cells both *in vitro* and *in vivo* and induced cell cycle arrest at G1/S phase and apoptosis. The prediction of genes potentially targeted by miRNA showed that miR-27b-5p and its downstream target YWHAZ were most significantly upregulated and the expression of both was negatively correlated. Further experiments verified that wogonin could exert anticancer effects by upregulating miR-27b-5p and downregulating YWHAZ (Ma et al., 2023b). In MHCC97L and HepG2 cells, wogonin *in vitro* and *in vivo* dose-dependently inhibited cell proliferation and induced G1-phase arrest, which was able to be reversed by GSK-3β knockdown without affecting apoptosis, suggesting that activation related to GSK-3β may dominate the process (Hong M et al., 2020).

#### 4.5.5 Wogonoside

Earlier studies reported that wogonoside inhibited the proliferation of Bel-7402 cells by inducing G2/M phase blockade and apoptosis (Li Y et al., 2015).

#### 4.5.6 *Oroxylin*-A

Non-steroidal anti-inflammatory drug activated gene-1 (NAG-1), one of the TGF-β, is thought to be associated with poor prognosis in many tumors (Lee J et al., 2019). Oroxylin-A not only directly inhibited the proliferation and migration of SMMC-7721, HepG2, and MHCC-97H cells, but also reversed the TGF-β-triggered rise in N-cadherin, Vimentin, and Twist1 and the decline in E-cadherin. Following experiment revealed that NAG-1 knockdown eliminated the inhibitory effect of oroxylin-A on TGF-β/pathway in SMMC-7721 cells, suggesting that oroxylin-A knockdown NAG-1 by upregulating it. In addition, animal experiments suggested a role for oroxylin-A in reducing SMMC-7721 lung metastasis, which could be blocked by NAG-1 knockdown or HDAC1 overexpression (Huo et al., 2022). PTEN induced kinase 1 (PINK) regulates tumor cell survival and chemotherapeutic drug resistance (Zheng F. et al., 2023). Cyclin-dependent kinase 9 (CDK9), a transcriptional activator belonging to the CDK family, whose inhibitor has been used in the treatment of a variety of tumors (Zhang Y. et al., 2024). Expression of CDK9 in HepG2, MHCC-97H, HLE, Huh7, and Hep3B cells was significantly higher than that in tumor cells HLE and normal cells LO2, and the CDK9 inhibitors LDC067 and PHA767491 inhibited the proliferation of HepG2 *in vitro* and *in vivo*, respectively. The time- and concentration-dependent inhibition of CDK9 viability in HepG2 cells by upregulation of wt-p53 and downregulation of p-MDM2, p-SIRT levels by oroxylin-A had a comparable effect to PHA767491 in animals *in vivo*. In addition, toxicological study *in vivo* showed that oroxylin-A has lower toxicity. This reveals that oroxylin-A safely exerts its therapeutic effect on HCC by restoring the function of wt-p53 and thus inhibiting CDK9 (Yao JY. et al., 2022). Similarly, LDC067-induced inhibition of CDK9 inactivated the SIRT3-FOXO3-BNIP3 axis and the PINK1-PRKN pathway as well, leading to disruption of mitochondrial homeostasis and cell death in HCC cells. Oroxylin-A downregulated SIRT3, FOXO3, BNIP3, PINK1, and PRKN through disruption of mitochondrial function. It is evident that oroxylin-A also reverses drug resistance in HCC cells by inhibiting mitochondrial autophagy mediated by the PINK-PRKN pathway (Yao J. et al., 2022). Hepatocyte nuclear factor 4α (HNF-4α), a positive regulator of HNF-1α downstream, is involved in hepatocyte differentiation (Morimoto A et al., 2017). The radio of pyruvate kinase M1 (PKM1) and pyruvate kinase M2 (PKM2) have been shown to be associated with metabolic alterations and proliferation of HCC cells (Li Y et al., 2023). Oroxylin-A inhibited proliferation and induced G2/M phase arrest of HepG2 and SMMC- 7721 cells. Furthermore, oroxylin-A induced cell differentiation accompanied by upregulation of ALB, HNF-4α and downregulation of PTB, AFP. Animal experiments showed that oroxylin-A inhibited tumor growth accompanied by protein changes consistent with that *in vitro*. It can be seen that oroxylin-A plays a therapeutic role by inducing primary HCC cell differentiation (Wei L et al., 2017). Oroxylin-A not only directly induced apoptosis in HepG2 and SMMC-7721 cells, but also, by altering extracellular vesicles released by HCC cells, promoted macrophage M1-like polarization. Moreover, oroxylin-A (300 mg/kg/d 2w) also enhanced the antitumor effects of PD-1/PD-L1 inhibitors in mice. Therefore, improving the tumor microenvironment and immune response of HCC is one of the mechanisms underlying the therapeutic effects of oroxylin-A (Wang P. et al., 2023).

Glucose transporter 1 (GLUT1) is used by malignant tumors to increase glucose utilization and is one of the prognostic markers of HCC (Kim H et al., 2024). Oroxylin-A induced apoptosis in HepG2 and SMMC-7721 cells, a process that was characterized by the downregulation of GLUT1 expression and significant reduction in ECAR, OCR, and ATP production. Experiments *in vivo* inhibited tumor growth and decreased the expression of SIRT1, PDK2, PARL1, MFN1, OPA1, YME1L1. It is evident that oroxylin-A inhibits HCC by limiting glucose metabolism and blocking mitochondrial fusion (Guo Y et al., 2023).

### 4.6 Pancreatic cancer

PC is known as the “king of cancers” due to its high mortality and aggressiveness, with a 5-year survival rate of less than 10% (Siegel RL et al., 2023). Currently, surgical resection is the only means of eradication of PC, which is increasingly resistant to mainstream anticancer drugs such as oxaliplatin (Kamisawa T et al., 2016; Perri G et al., 2020). The direct and indirect therapeutic effects of flavonoids of *S. baicalensis* on PC are shown in (Table 6).

**TABLE 6:** Flavonoids of *Scutellaria baicalensis* in the treatment of PC.

Name	Dose	Subjects	Mechanism	Effect	Reference
Baicalein	50, 75, 100 μmol/L	BxPC-3 and PANC-1 cells	caspase-3↑Bax↑cyclinD1↓cyclinE↓cyclinA↓Bcl-2↓	Inhibit proliferation Promote autophagy and apoptosis	Ao P et al. (2018)
	25 mg/kg/d	PANC, HM-SUIT-2 cells; HM-SUIT-2 cells xenograft mice	FGFBP1↓	Inhibit proliferation and liver metastasis	Zhang C et al. (2023)
	50, 100 M 72h10 mg/kg/tiw ip	PANC-1 cells xenograft BALB/c mice	miR-139-3p↑ING5↑miR-196b-5p↓NOB1↓	Inhibit proliferation, migration and invasion Promote apoptosis Induce cell cycle arrest at S phase	Ma D et al. (2021)
	2.5, 5, 10, 20, 40, 80, 160, 320 μM	CAPAN2 and HTRET-HPNE cells	cleaved caspase-3↑Bax↑caspase-3↓Bcl-2↓	Inhibit viability Promote apoptosis Induce cell cycle arrest at S phase	Zhang Y et al. (2020)
	2, 4, 8, 16, 32, 64, 128, 256 μM 24, 48, 72h; 20, 60 mg/kg/d ig	PANC-1 cells xenograft BALB/c mice	CD44↓CD24↓Oct-4↓Sox-2↓Gli-2↓	Inhibit proliferation and migration	Song L et al. (2018)
	25, 50, 75, 100 μM	BxPC-3, PANC-1, HL-7702, 293T cells	Bax↑cleaved caspase-9↑p21↑p27↑PDK1↓	Inhibit proliferation and migration Promote apoptosis Induce cell cycle arrest at G0/G1 phase	Zhou RT et al. (2017)
	0.1, 0.2, 0.4, 0.8, 1.6, 3.2, 6.25, 12.5, 25, 50, 100 μM	CFPAC-1 cells; CFPAC-1 cells xenograft BALB/c mice	Bax↑caspase-8↑PARP↑Bcl-2↓survivin↓	Inhibit viability and proliferation Promote apoptosis Increase sensitivity to gemcitabine	Li et al. (2018a)
Baicalin	40, 80, 120, 160 μmol/L	SW1990 cells	p15↑Bax↑ cleaved caspae-8↑p53↑CDK2↓Bcl-2↓	Inhibit proliferation, migration and invasion Promote apoptosis	Huang Q et al. (2019)
Wogonin	5, 10, 20, 40, 60, 80, 100 M; 60 mg/kg/d ip	PANC-1 and AsPC-1 cells; PANC-1 cells xenograft BALB/c mice	Fe^2+^↑TF↑TFRC↑ROS↑GSH↓Nrf2↓GPX4↓HO-1↓SLC7A11↓	Promote ferroptosis	Liu et al. (2023b)
	1.3,2.5,10,20,40,80,160 μM; 50 mg/kg/d ig	PANC-1, BXPC-3, PANC-02 cells; PANC-02 cells xenograft C57BL/6 mice	Bad↑p-Akt↓Bcl-2↓	Inhibit proliferation Promote apoptosis Increase sensitivity to gemcitabine	Zhang T et al. (2022)
Wogonoside	1.5, 3, 6.25, 12.5, 25, 50, 100, 200, 400, 800μM; 80 mg/kg/d ig	PANC-1 and SW1990 cells; PANC-1xenograft BALB/c mice	PCNA↓p21↓CD44↓SOX2↓N-cad↓MMP-14↓IL-6↓TNF-Iα↓L-1β↓	Inhibit viability and proliferation Promote apoptosis	Huang H et al. (2021)

#### 4.6.1 Baicalein

It is previously reported that baicalein (50–100 mol/L 48 h) was able to upregulate apoptosis-related genes caspase-3 and Bax, downregulate the protein expression of cell cycle genes cyclinD1, cyclinE, cyclinA, and apoptosis gene Bcl-2, and concentration-dependently inhibit proliferation of BxPC-3 and PANC-1 cells and promoted autophagy and apoptosis in PC cells. Meanwhile, 75 mol/L baicalein and 20 mol/L had similar *in vitro* inhibitory effects (Ao P et al., 2018). Cancer-associated fibroblasts (CAF) is the most prominent cell within the PC stromal, favoring tumor progression (Niu N et al., 2024). Experiment has shown that baicalein inhibits PANC and HM-SUIT2 cell viability accompanied by downregulation of FGFBP1 gene expression. In addition, baicalein suppressed the growth and liver metastasis of HM-SUIT2 in xenograft mice. It suggests that baicalein improves the tumor of PDAC through CAF microenvironment (Zhang C et al., 2023).

The concentration-dependent induction of S-phase cell cycle arrest and apoptosis by baicalein was accompanied by an increase in p21 levels and a decrease in CCND1 levels, having an inhibitory effect on the proliferation, motility, and invasion of PANC-1 cells, which was identified in the animal experiments. Further studies revealed that this process was accompanied by the upregulation of 20 miRNAs, of which miR-139-3p is the most abundant, and the downregulation of 39 miRNAs, of which miR-196b-5p is the most abundant, promoting apoptosis by up-regulating ING5 and down-regulating NOB1 expression (Ma D et al., 2021). Cisplatin-resistant PC cell CAPAN2 showed concentration-dependent viability inhibition and S-phase cell cycle arrest after baicalein treatment, compared to normal pancreatic cells HTRET-HPNE. This process was accompanied by an increase in cleaved caspase-3, Bax expression and a decrease in caspase-3, Bcl-2 expression, suggesting that baicalein also has a selective pro-apoptotic effect on PC-resistant cells (Zhang Y et al., 2020). Activation of the sonic Hedgehog (Shh) signaling pathway in cancer stem cell (CSC) is closely associated with PC (Jeng KS et al., 2020). Baicalein not only directly inhibits PANC-1 cell proliferation and migration, but also affects sphere formation of pancreatic CSCs. Further experiment revealed that baicalein treatment decreased the expression of CSC markers CD44, CD24, Oct-4, Sox-2 and effector Gli-2. Animal experiments also showed that tumor growth inhibition in xenograft mice treated by baicalein was accompanied by downregulation of Shh pathway and expression (Song L et al., 2018). The level of neural precursor cell expressed developmentally downregulated 9 (NEDD9), a scaffold protein in focal adhesions, correlated with PC cell migration, invasion, and metastasis (Kondo S et al., 2012). Baicalein dose- and time-dependently inhibited the proliferation and migration of BxPC-3 and PANC-1 cells, and induced G0/G1 phase arrest, which was consistent with the effects of the PI3K inhibitor LY294002 and the MEK inhibitor PD98059. Upregulation of Bax, cleaved caspase-9, p21, p27 levels and downregulation of PDK1 also occurred. Notably, the anticancer ability of baicalein was instead decreased at high concentrations. Following studies revealed that NEDD9 knockdown induced tumor cell apoptosis by inhibiting p-ERK, p-Akt expression, which was consistent with the effect of baicalein, and its overexpression reversed this trend. It can be seen that baicalein exerts its anti-PC effect by inactivating PI3K/Akt and MEK/ERK by reducing NEDD9 expression (Zhou RT et al., 2017).

Earlier studies demonstrated that baicalein (3.2–100 μM 48 h) synergized with gemcitabine to concentration-dependently inhibit CFPAC-1 and PANC-1 cell viability. Baicalein also synergized with gemcitabine to inhibit tumor growth in xenograft mice *in vivo*. The combination of them showed intracellular rise in Bax, caspase-8, PARP and decrease in Bcl-2, survivin, proving that the pro-apoptotic effect is an important component of the therapeutic effect (Li Z. et al., 2018).

#### 4.6.2 Baicalin

Proliferation, migration and invasion of SW1990 cells were inhibited by baicalin in a dose-dependent manner, which was accompanied by p15, ROS, p-JNK, Foxo1, BIM upregulation and CDK2 downregulation and would be reversed by the free radical scavenger NAC. It is reasonable to hypothesize that baicalin induces apoptosis by activating the JNK/Foxo1/BIM pathway to inhibit PC (Huang Q et al., 2019).

#### 4.6.3 Wogonin

Wogonin time- and dose-dependently induced PANC-1 and AsPC-1 Cell death, accompanied by an increase in Fe^2+^, TF, TFRC, and ROS, which is reversed by the ferroptosis inhibitors DFO or Fer-1. Treatment of animals showed the same effect without organ toxic effects. It is evident that wogonin counteracts PAAD by inducing iron death and lipid peroxidation (Liu X. et al., 2023).

Furthermore, wogonin inhibited the growth of gemcitabine-resistant cells PANC-1 and increased cellular sensitivity to the drug *in vitro*. Further studies revealed that wogonin promotes apoptosis through upregulation of Bad and downregulation of p-Akt, Bcl-2 during this process. Oral administration also led to the same changes in animals, showing that wogonin indeed reverses PC cell gemcitabine-resistance by inhibiting the Akt pathway (Zhang T et al., 2022).

#### 4.6.4 Wogonoside

Tumor necrosis factor receptor-associated factor 6 (TRAF6) is widely involved in PC cell growth and migration (Rong et al., 2014). Experiments *in vitro* demonstrated that Wogonoside concentration-dependently decreased viability and promoted apoptosis of PANC-1 and SW1990 cells, which was accompanied by downregulation of proliferation-associated proteins (PCNA and p21), stem cell marker proteins (CD44 and SOX2)and mesenchymal transition marker proteins (N-cad and MMP-14). In addition, wogonoside reduced the levels of IL-6, TNF-α, IL-1β and reversed tumor growth caused by overexpression of TRAF6 and its downstream proteins *in vivo*. It is evident that wogonoside counteracts PC by inhibiting the TRAF6-mediated tumor microenvironment (Huang H et al., 2021).

## 5 Disscusion

The 5 flavonoids mentioned above are the most intensively studied ingredients of *S. baicalensis* by far. Numerous preclinical studies have shown that the pharmacological effects of *S. baicalensis* and its flavonoids are realized through multiple pathways such as, making their pharmacological effects more diverse and potential compared with drugs functioning through single pathway, which have not yet been fully revealed. A summary of the research conducted in the last decade shows that, although in its infancy, the use of *S. baicalensis* in the treatment of digestive system tumors, in particular GC and HCC, is on the rise, and the quality of the trials has improved significantly. Most studies have demonstrated exciting effects of *S. baicalensis* taken orally or flavonoids injected, and that these effects are synergistic with other treatment methods without significant toxicity. Meanwhile, a growing body of research is making it possible to utilize *S. baicalensis* and its flavonoids more efficiently, including the production of the herb, the extraction of the flavonoids, and the mode of administration. For example, overexpression of transcription factor SbMYB3 was investigated to increase the accumulation of flavonoid components, creating an opportunity for the production of more active ingredients (Fang Y et al., 2022). Chilling treatment (4°C 2–8 d) increased the concentration of flavonoids, including baicalin, baicalein, and wogonin, in the root of *S. baicalensis* Georgi as well (Yeo HJ et al., 2022). In addition, the optimized ultrasound-assisted enzymatic pretreatment process was able to significantly improve the extraction efficiency of baicalein and wogonin (Yun et al., 2022). In addition, the synthesis of baicalein and wogonin and their related derivatives is becoming more mature, and larger scale production is coming into reality (Zhao Z et al., 2022). pH-responsive sodium alginate/polyaspartate/CaCO_3_ *in situ* hydrogel with sustained release behavior and outstanding biodegradability significantly prolonged the residence time of baicalin in the stomach, which is also a potential oral drug delivery system (Xu L et al., 2023). These results provide us with solutions to the material basis for the application of *S. baicalensis*.

However, it is troubling that although Scutellaria baicalensis has been used clinically in China for the treatment of digestive system tumors by direct decoction in water and oral administration, the extraction and application of its bioactive ingredients like flavonoids have not yet come out of the laboratory. For example, due to practical constraints, most of these studies have been limited to elucidating the possible role of a single mechanism in the pharmacological effects on tumor cells, which is still a huge gap from the clinical use of drugs and bioactive ingredients. The minority of synergistic studies with anticancer therapies also lacked further exploration of the mechanisms. An animal study found that baicalein was best tolerated in mice compared to four other anti-tumor active ingredients of TCM (curcumin, betulinic acid, resveratrol and dihydroartemisinin), which may give a side note on its safety in the same drug class (Gao et al., 2024). Another recent review also illustrates the insignificant toxicity of baicalein (Lei C et al., 2024). This low toxicity is also shown for wogonoside and wogonin (Yan Y et al., 2020; Tong Y et al., 2024). Although sufficient evidence is lacking, it is reasonable to hypothesize that the flavonoids of *S. baicalensis* have acceptable toxicity based on these studies. Consequently, in the future, research and application of *S. baicalensis* and its flavonoids should focus on the following points: (1) Transfer to clinical studies gradually to prepare for the clinical studies of novel formulations, in particular, the effect of body metabolism on the distribution of flavonoids; (2) Upgrading cultivation and extraction techniques to produce herbs and extracts more steadily; (3) Paying more attention to the cooperation between drugs. On the one hand, the cooperation of many kinds of TCMs can give the advantage of multi-targets, and many TCM prescriptions with a long history can function well as reference in this regard; on the other hand, the combination of *S. baicalensis* or its flavonoids with modern chemotherapeutic drugs has been proved to have a synergistic effect without any obvious adverse reaction, and the combination therapy of traditional herbs and modern drugs is a potential solution. Combination therapy of traditional herbs with modern drugs is a promising direction.

In conclusion, our study demonstrated that *S. baicalensis* and its flavonoids have great potential in the treatment of various digestive tumors and are worthy of further study and application.

## Glossary

12-LOX: 12-lipoxygenase
BTG3: B-cell translocation gene 3
BLVRA: Biliverdin reductase A
CAF: Cancer-associated fibroblasts
CAT: catalase
CDK: cyclin-dependent kinase
DEPP: decidual protein induced by progesterone
MMR: DNA-mismatch repair
EGFR: epidermal growth factor receptor
EC: esophageal cancer
EAC: esophageal adenocarcinoma
ESCC: esophageal squamous cell carcinoma
FAS: factor associated suicide
FASL: factor associated suicide ligand
FAK: focal adhesion kinase
GC: gastric cancer
Gli1: Glioma
GPX4: glutathione peroxidase 4
GSK-3β: glycogen synthase kinase 3-β
GLUT1: glucose transporter 1
GADD45a: growth arrest and DNA damage-inducible 45a
HSP: heat shock protein
HCC: hepatocellular carcinoma
HK2: hexokinase-2
HIF-1α: hypoxia inducible factor-1α
IRF: interferon regulatory factor
ICC: intrahepatic cholangiocarcinoma
IREB2: iron-responsive element binding protein 2
LDHA: lactate dehydrogenase A
LATS1: large-tumor suppressor kinase 1
MMP: matrix metalloproteinase
MET: mesenchymal epithelial transition factor
miRNA: microRNAs
MCT-4: monocarboxylate transporter-4
NEDD9: neural precursor cell expressed developmentally downregulated 9
NAG-1: non-steroidal anti-inflammatory drug activated gene-1
OSCC: oral squamous cell carcinoma
PAK4: p21-activated kinase 4
PC: pancreatic cancer
PI3K: phosphatidylinositol 3-kinase
PD-L1: programmed cell death-ligand 1
PD1: programmed cell death protein 1
Akt: protein kinase B
PINK: PTEN induced kinase 1
PDK1: pyruvate dehydrogenase kinase-1
PKM: pyruvate kinase M
SUFU: serine/threonine kinase
SMO: smoothened
SHH: sonic hedgehog
Sp1: Specificity protein 1
ROS: reactive oxygen species
SOD: superoxide dismutase
SLC7A11: synthetase long chain family member 4
TRAIL: TNF-related apoptosis-inducing ligand
TAZ: transcriptional co-activator with PDZ-binding motif
TGF-B: transforming growth factor-B
TICs: tumor initiating stem cell-like cells
TRAF6: tumor necrosis factor receptor-associated factor 6
ULK1: unc51-likekinase 1
YAP: Yes-related protein
